# Respiratory admissions and impact of COVID‐19 lockdowns for children with severe cerebral palsy

**DOI:** 10.1111/dmcn.16346

**Published:** 2025-05-08

**Authors:** Susan M. Reid, Moya Vendeleur, Danielle Wurzel, Katherine Frayman, Joshua Osowicki, Kylie Crompton, Gordon Baikie, Giuliana Antolovich, Angela Guzys, Monica S. Cooper

**Affiliations:** ^1^ Neurodisability and Rehabilitation Murdoch Children's Research Institute Parkville Victoria Australia; ^2^ Department of Paediatrics University of Melbourne Parkville Victoria Australia; ^3^ Department of Neurodevelopment and Disability The Royal Children's Hospital Parkville Victoria Australia; ^4^ Department of Respiratory and Sleep Medicine The Royal Children's Hospital Parkville Victoria Australia; ^5^ Respiratory Diseases Group Murdoch Children's Research Institute Parkville Victoria Australia; ^6^ Allergy and Lung Health Unit, School of Population and Global Health University of Melbourne Parkville Australia; ^7^ Tropical Diseases Research Group Murdoch Children's Research Institute Parkville Victoria Australia; ^8^ Department of Infectious Diseases The Royal Children's Hospital Parkville Victoria Australia

## Abstract

**Aim:**

To explore factors contributing to the burden of respiratory admissions in children with severe cerebral palsy (CP) by comparing admissions to a single tertiary paediatric hospital before, during, and after the period of social restrictions implemented to reduce transmission of COVID‐19 (lockdown period).

**Method:**

For this observational study, three severe CP cohorts (pre‐lockdown, lockdown, post‐lockdown) were identified from a state‐wide cerebral palsy register and linked to patient‐level clinical and demographic data. Medical records were manually searched to identify respiratory hospitalizations. Frequency and details of admissions were compared across the three 2‐year periods.

**Results:**

During the lockdown period, there were 24 hospitalizations for respiratory illness per 100 children compared to 37 and 47 in the pre‐lockdown and post‐lockdown periods (*p* < 0.001). Respiratory viruses (excluding picornaviruses) were detected in only 7% of lockdown admissions compared to 24% pre‐lockdown and 30% post‐lockdown. Sputum sampling was performed in 34% of admissions with gram‐positive bacteria cultured in 6% admissions and gram‐negative bacteria only in 18%.

**Interpretation:**

The study findings highlight an important dynamic contribution of viral infections to respiratory illnesses in children with severe CP and the potential to improve outcomes with personalized approaches based on defining individual factors predisposing to recurrent respiratory admissions.

## Graphical abstract

In a population cohort of children with severe cerebral palsy, there was a reduced frequency of hospitalization for respiratory illness during periods of restricted social contact, implemented to reduce transmission of COVID‐19. This mirrored trends in the wider population and underlines the importance of community viral transmission in these children.

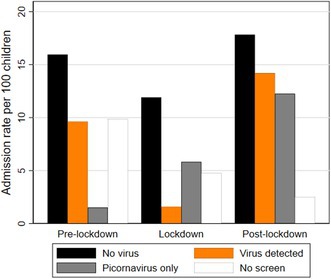

Plain language summary: https://onlinelibrary.wiley.com/doi/10.1111/dmcn.16409

**This original article is commented on by Paneth on pages** 1522‐1523 **of this issue.**

**What this paper adds**

- Community‐acquired viral pathogens are common triggers for respiratory exacerbations in severe cerebral palsy.
- Gram‐positive bacterial growth in sputum was reported in 6% of admissions.
- In 18% of admissions, only gram‐negative bacteria (e.g. *Pseudomonas aeruginosa*) were cultured.
- The precipitants for respiratory exacerbations, and drivers for management decisions, are incompletely understood.

Cerebral palsy (CP) describes a diverse group of conditions characterized by impairment of motor function and limitations in performing activities of daily living. By definition, these conditions are non‐progressive and include cerebral maldevelopments, malfunctions, and destructive insults before 2 years of age. CP occurs in approximately 1.4 per 1000 live births in Australia.^1^ This equates to approximately 110 children with CP per birth year in the Australian state of Victoria, including approximately 30 with severe motor impairment. Approximately 30 additional persons with CP per birth year immigrate to Victoria.

Children with severe CP have more frequent and severe acute lower respiratory tract infections and chronic lung disease relative to other children,^2^ generally attributed to poor postural control, ineffective cough, kypho‐scoliosis,^3^ and poor oropharyngeal function.^4^ Swallowing difficulties and gastro‐oesophageal reflux can further complicate airway management and nutrition.^5^ Malnutrition and chronic airway infection by pathogenic bacteria may contribute to recurrent episodes of acute respiratory tract infection and slowly progressive lung disease.^6^ The compounding effects are a major burden on the children, their families, and the health care system.^7^, ^8^ In Australia, respiratory illness is the most common reason for admission amongst children with CP functioning in Gross Motor Function Classification System (GMFCS) level V,^7^ and the leading cause of unplanned hospitalization in children with CP, contributing to 36% of all emergency admission costs.^9^

Although community‐acquired lower respiratory tract infections in childhood are most often viral and self‐limiting, they are also an important cause of serious illness, hospitalization, intensive care unit admission, and death. Children with severe CP are overrepresented amongst those with critical illness.^10^ It remains uncertain the extent to which hospital admissions for these children follow patterns of community‐acquired infections or are predominantly the result of the multitude of structural and functional impairments associated with severe CP.

The impact of personal and population‐level measures to reduce COVID‐19 transmission in 2020 and 2021 affected transmission of other viral pathogens and the incidence of related respiratory diseases.^11^, ^12^, ^13^, ^14^, ^15^ These events provide an unusual opportunity to better understand the relative contribution of different factors to the burden of respiratory admissions in children with severe CP. In this study, we aimed to compare the frequency and details of admissions for children with severe CP to a single tertiary paediatric hospital before, during, and after the period when Victoria implemented many and varied population‐level interventions to reduce COVID‐19 transmission, including prolonged restrictions on human movement and contact, commonly described as ‘lockdowns’.

## METHOD

### Study setting

The study was conducted at The Royal Children's Hospital, Melbourne and was approved by the hospital's Human Research Ethics Committee as part of the Victorian Cerebral Palsy Register project (HREC #91020). As part of the enrolment process for the Register project, families provided consent for collection of clinical data from medical records.

### Study periods

Data for the study were collected for the period 1st April 2018 to 31st March 2024. Melbourne residents experienced 263 days of lockdown restrictions between 31st March 2020 and 27th October 2021. Additional measures such as mask‐wearing and physical distancing were implemented more broadly over the same period. After the period of lockdowns, there was a shoulder period of slowly increasing social freedoms. To capture data for periods of the same length, for this study, the lockdown period was designated as 1st April 2020 to 31st March 2022, the pre‐lockdown period as 1st April 2018 to 31st March 2020, and the post‐lockdown period as 1st April 2022 to 31st March 2024.

### Study cohorts and eligibility criteria

Three cohorts (pre‐lockdown, lockdown, and post‐lockdown) were identified from the Victorian Cerebral Palsy Register database as the basis for ascertaining respiratory admissions during the corresponding study period. Children were included in each cohort if (1) consent had been provided to participate in the Register project, (2) their gross motor function was classified as GMFCS levels IV or V, (3) they were aged between 3 years and 16 years at the start of the study period, and (4) they were living in Victoria for the entire 2‐year period. Children who died, migrated, or turned 19 years during each study period were excluded from that cohort. This methodology meant that a single child could appear in more than one cohort and that, although the cohorts were matched for age, they included different participants at the lower and upper limits of age and based on residency status.

### Data collection and management

Clinical and demographic data on each participating child were obtained from the Victorian Cerebral Palsy Register database. Each child's electronic medical record at The Royal Children's Hospital was manually searched to identify all hospitalizations for a lower respiratory tract infection or exacerbation of chronic respiratory disease during the 2‐year pre‐lockdown, lockdown, and post‐lockdown periods. Inclusion was based on the recorded discharge diagnosis, irrespective of time between admissions. The number and details of admissions were recorded for each period where eligibility criteria were met. Sputum culture data were collected and broadly classified as not done, no significant finding, or pathogen isolated. Types of pathogens were recorded together with the presence and quantity of gram‐negative bacilli such as *Pseudomonas aeruginosa*. Results of respiratory molecular panel tests (AusDiagnostics respiratory pathogen panel 16‐well [REF 20620]) of nasopharyngeal swabs for common viruses were collected if performed within 5 days of the admission date. Viruses covered by the panel were SARS‐CoV‐2, influenza A and B, respiratory syncytial virus A and B, parainfluenza virus 1–4, metapneumovirus A and B, adenovirus A–E, rhinovirus and enterovirus, enterovirus, and parechovirus. Admissions were categorized on whether a picornavirus (rhinovirus or enterovirus) was the sole finding, a virus other than picornavirus was detected, or no pathogens were detected. Admissions for COVID‐19 infections were excluded as they represented an exposure not present in the pre‐lockdown period and potentially having a lower threshold for admission.

### Statistical methods

All tabulations and statistical analyses were conducted using Stata version 17.1 (StataCorp, College Station, TX, USA). Numerical data related to admissions were not normally distributed and were converted to categorical variables for comparison between periods. Missing data were excluded if below 5%; otherwise, missing data were reported in tabulations.

## RESULTS

### Study cohorts

**FIGURE 1:**
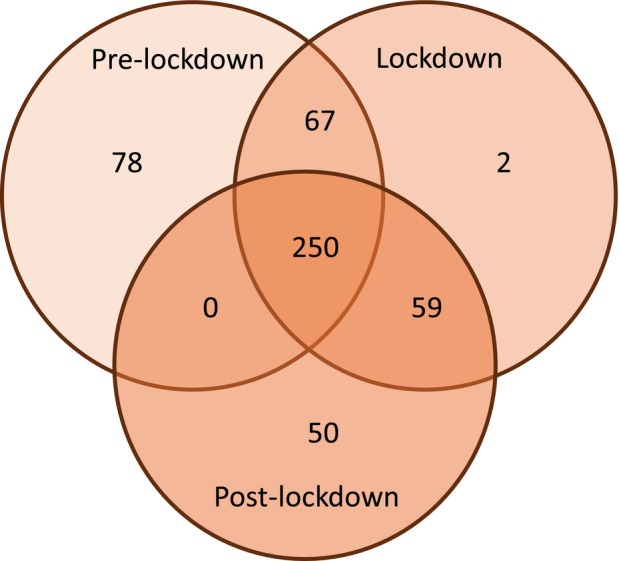
Venn diagram showing the number of children in each cohort and overlap between cohorts.

From a total of 506 children, 395, 378, and 359 children were included in the pre‐lockdown, lockdown, and post‐lockdown cohorts respectively (Figure S1). Compared to the pre‐lockdown cohort, the overlapping proportions of children in the lockdown and post‐lockdown cohorts were 84% and 70% respectively (Table 1 and Figure 1). Fewer children died during the lockdown period compared to the pre‐ and post‐lockdown periods. There was also a progressive decrease in the proportion of children in each cohort with a feeding tube or fundoplication (Table 1). Apart from those differences, characteristics of children were similar across periods.

**TABLE 1:** Comparison of characteristics of children in the pre‐lockdown, lockdown, and post‐lockdown cohorts.

	Pre‐lockdown *n* = 395	Lockdown *n* = 378	Post‐lockdown *n* = 359	Missing data (%)
Overlap	Reference	317 (83.9)	250 (69.6)	
Deaths during period (excluded)	19	14	22	0
Male sex	228 (57.7)	215 (56.9)	204 (56.8)	0
Age 9–16 years at period start	228 (57.7)	207 (54.8)	197 (54.9)	0
Born outside Victoria	75 (19.0)	74 (19.6)	70 (19.5)	0
Birth gestation 35+ weeks	259 (67.4)	248 (68.5)	241 (69.6)	3.6
GMFCS level V	183 (46.3)	175 (46.3)	172 (47.9)	0
Non‐spastic/mixed motor disorder	289 (73.5)	283 (75.5)	270 (76.1)	0.8
Quadriplegic pattern	351 (90.9)	334 (90.8)	317 (90.8)	2.8
Epilepsy diagnosis	237 (60.9)	222 (59.7)	211 (59.8)	1.6
Intellectual disability	333 (87.0)	312 (87.4)	290 (87.9)	6.1
Feeding tube in situ	172 (43.6)	152 (40.4)	137 (38.4)	0
Fundoplication surgery	44 (11.2)	30 (8.0)	24 (6.7)	0

*Note*: Data are *n* (%) unless otherwise indicated. Abbreviation: GMFCS, Gross Motor Function Classification System.

Overall, the 506 eligible children had 407 admissions, a median of 0 (interquartile range 0–14) admissions per child. Only eight hospitalized children had previous chest computed tomography (CT) and three of these had a CT‐confirmed diagnosis of bronchiectasis. Home non‐invasive ventilation support was administered for 37 children for varying lengths of time during the study period.

### Admissions for respiratory illness across periods

Excluding nine admissions for COVID‐19 during the lockdown period and 20 post‐lockdown, admissions decreased during the lockdown period and increased in the post‐lockdown period to exceed pre‐lockdown results (Table 2). The number of admissions decreased by a factor of 0.62 (95% confidence interval [CI] 0.48, 0.81) between the pre‐lockdown and lockdown periods and increased by a factor of 1.87 (95% CI 1.45, 2.41) between the lockdown and post‐lockdown periods (*p* < 0.001). Admissions per 100 children were 37, 24, and 47 for the three periods respectively (*p* < 0.001 for difference across periods).

**TABLE 2:** Admissions and related characteristics across the 2‐year pre‐lockdown, lockdown, and post‐lockdown periods.

	Pre‐lockdown	Lockdown	Post‐lockdown
*Admissions*			
Total admissions, *n*	146	91	170
Children with at least one admission	57 (14.4)	38 (10.0)	59 (16.4)
Children with multiple admissions	27 (6.8)	23 (6.1)	37 (10.3)
Admissions/100 children	37.0	24.1	47.4
Total admitted days	1153	684	1465
*Severity indicators*			
Length of admission			
1–3 days	53 (36.3)	40 (44.0)	66 (38.8)
4–9 days	56 (38.4)	35 (38.5)	62 (36.5)
10+ days	37 (25.3)	16 (17.6)	42 (24.7)
Length of intensive care admission			
0 days	102 (69.9)	71 (78.0)	123 (72.3)
1–6 days	23 (15.7)	13 (14.3)	28 (16.5)
7+ days	21 (14.4)	7 (7.7)	19 (11.2)
Non‐invasive ventilation in intensive care			
0 hours	107 (73.3)	71 (78.0)	127 (74.7)
1–99 hours	20 (13.7)	10 (11.0)	24 (14.1)
100+ hours	19 (13.0)	10 (11.0)	19 (11.2)
Physiotherapy visits			
0	29 (19.9)	25 (27.5)	45 (26.5)
1–4	31 (21.2)	30 (33.0)	40 (23.5)
5–13	32 (21.9)	21 (23.1)	42 (24.7)
14+	54 (37.0)	15 (16.5)	43 (25.3)
Chest X‐rays performed			
0	14 (9.6)	12 (13.2)	12 (7.1)
1	85 (58.2)	56 (61.5)	112 (65.9)
2+	47 (32.2)	23 (25.3)	46 (27.1)
*Laboratory investigations*			
Respiratory molecular panel			
No virus detected	64 (43.8)	45 (49.4)	64 (37.6)
Virus detected (excl picornavirus only)	34 (23.3)	6 (6.6)	51 (30.0)
Picornavirus detected as sole finding	5 (3.4)	22 (24.2)	44 (25.9)
No testing performed	43 (29.5)	18 (19.8)	11 (6.5)
Sputum culture			
Nil significant	19 (13.0)	8 (8.8)	18 (10.6)
Gram‐positive bacteria	11 (7.5)	6 (6.6)	6 (3.5)
Gram‐negative bacteria only	27 (18.5)	14 (15.4)	31 (18.2)
No testing performed	89 (61.0)	63 (69.2)	115 (67.7)
C‐reactive protein			
< 50mg/L	55 (37.7)	44 (48.3)	71 (41.8)
≥ 50mg/L	29 (19.9)	16 (17.6)	40 (23.5)
No testing performed	62 (42.5)	31 (34.1)	59 (34.7

*Note*: Data are *n* (%) unless otherwise indicated.

Despite the increasing mean age and potential worsening of chronic lung disease over time, admissions for the 250 children included in all three cohorts (Figure 1) followed trends for the wider groups, hospitalized on 78, 57, and 102 occasions respectively, over the pre‐lockdown, lockdown, and post‐lockdown periods.

#### Severity indicators

Fewer physiotherapy treatments were performed during the lockdown period but there was a lack of strong evidence for any differences between periods in length of admission, time spent in the intensive care unit, use of non‐invasive ventilation in intensive care, and number of chest X‐rays performed (Table 2).

#### Respiratory molecular panel

**FIGURE 2:**
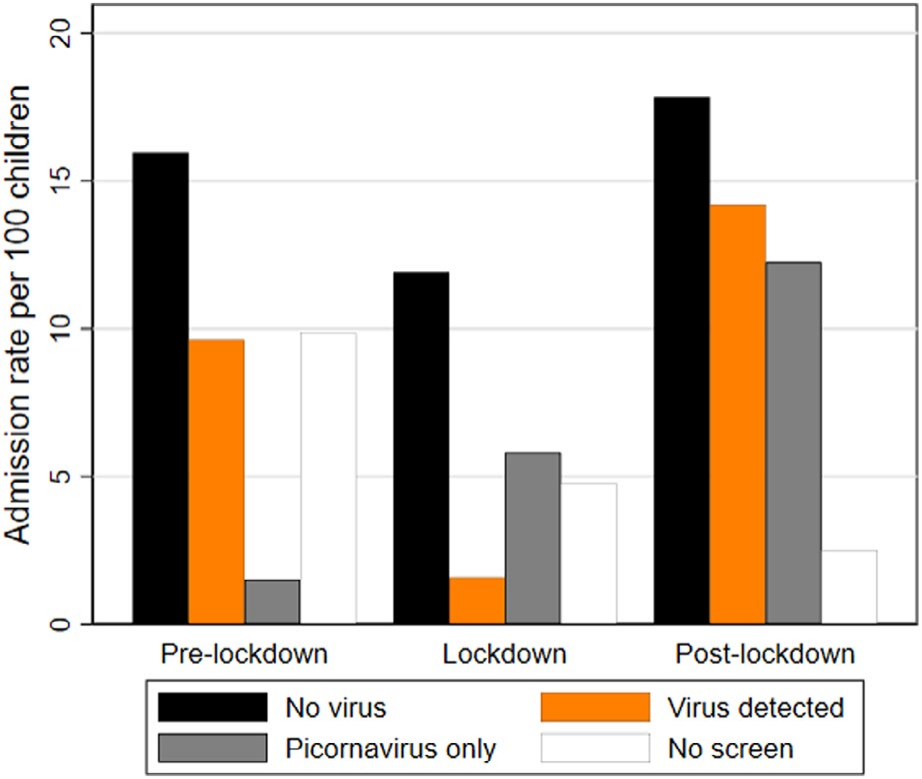
Admissions for respiratory illness per 100 children by viral status across pre‐lockdown, lockdown, and post‐lockdown periods (excluding admissions for COVID‐19).

Across periods, there was strong evidence of difference in testing frequencies and test results (*p* < 0.001). The proportion of admissions in which respiratory molecular tests were done increased over time (Table 2). There was also a substantial reduction during the lockdown period in the number of admissions, the proportion of the cohort admitted, and the number of admissions per 100 children where a respiratory virus, other than picornavirus, was detected from 34 (23%; 8.6 per 100 children) to 6 (7%; 1.6 per 100 children). Conversely, admissions in which a picornavirus (rhinovirus and/or enterovirus) was detected increased in the lockdown period from 5 (3%; 1.5 per 100 children) to 22 (24%; 5.8 per 100 children). Post‐lockdown, admissions with diagnosed viral infections increased beyond the levels seen during the previous two periods to 12.2 admissions per 100 children for picornaviruses and 14.2 for other respiratory viruses (Table 2 and Figure 2).

#### Sputum culture

Sputum culture was done in 140 (34%) admissions, with more frequent testing during admissions to intensive care (59% vs 25%). A pathogen was identified from 95 (68%) cultures, corresponding to 23% of all admissions (Table S1). Gram‐negative bacterial growth was the sole finding in 72 admissions, that is, in 6% of all admissions and 76% of all positive cultures. There was little difference between periods in the proportion of admissions where sputum culture was done, the proportion with a positive culture, or the proportion where only gram‐negative bacteria were cultured (Table 2).

#### C‐reactive protein

C‐reactive protein testing patterns and results were similar between periods (Table 2).

### Viral testing over the whole 6‐year period

Respiratory molecular panel tests were done in 82% of admissions, with a virus detected in half of these. Viruses are listed in Table S1. There was a lower likelihood of respiratory virus testing during shorter admissions of 1 to 3 days where intensive care unit admission was not required (Table 3). Conversely, the longest hospital and intensive care admissions were associated with negative viral tests.

**TABLE 3:** Admission characteristics based on viral status.

	No virus *n* = 173	Viral RNA detected, excl. picornavirus only *n* = 91	Picornavirus only *n* = 71	No screen *n* = 72	Total *n* = 407	*p*
Sputum culture						0.079
Nil significant	20 (11.6)	8 (8.8)	9 (12.7)	8 (11.1)	45 (11.1)	
Gram‐positive	9 (5.2)	4 (4.4)	3 (4.2)	7 (9.7)	23 (5.6)	
Gram‐negative only	43 (24.9)	13 (14.3)	10 (14.1)	6 (8.3)	72 (17.7)	
Not done	101 (58.4)	66 (72.5)	49 (69.0)	51 (70.8)	267 (65.6)	
Antibiotics						0.025
Not used	11 (6.4)	16 (17.6)	11 (15.5)	7 (9.7)	45 (11.1)	
Used	162 (93.6)	75 (82.4)	60 (84.5)	65 (90.3)	362 (88.9)	
Days on antibiotics						0.273
0	11 (6.4)	16 (17.6)	11 (15.5)	7 (9.7)	45 (11.1)	
1–7	88 (50.9)	45 (49.4)	33 (46.5)	33 (45.8)	199 (48.9)	
8+	63 (36.4)	27 (29.7)	23 (32.4)	28 (38.9)	141 (34.6)	
Missing	11 (6.4)	3 (3.3)	4 (5.6)	4 (5.6)	22 (5.4)	
Length of stay						0.696
1–3	63 (36.4)	36 (39.6)	26 (36.6)	34 (47.2)	159 (39.1)	
4–9	69 (39.9)	36 (39.6)	25 (35.2)	23 (31.9)	153 (37.6)	
10+	41 (23.7)	19 (20.9)	20 (28.2)	15 (20.8)	95 (23.3)	
Days in ICU						0.078
0	114 (65.9)	66 (72.5)	54 (76.1)	62 (86.1)	296 (72.7)	
1–6	35 (20.2)	15 (16.5)	9 (12.7)	5 (6.9)	64 (15.7)	
7+	24 (13.9)	10 (11.0)	8 (11.3)	5 (6.9)	47 (11.6)	
C‐reactive protein						< 0.001
< 50mg/L	89 (51.4)	29 (31.9)	20 (28.2)	32 (44.4)	170 (41.8)	
≥ 50mg/L	29 (16.8)	30 (33.0)	21 (29.6)	5 (6.9)	85 (20.9)	
Not performed	55 (31.8)	32 (35.2)	30 (42.2)	35 (48.6)	152 (37.3)	

*Note*: Data are *n* (%) unless otherwise indicated.

Abbreviations: ICU, intensive care unit; RNA, ribonucleic acid.

Of 162 admissions where a virus was detected within the first 5 days, only 47 also had sputum culture performed. Of those 47 admissions, there was a positive culture in 30, with all but three of these including gram‐negative organisms such as *Pseudomonas aeruginosa*. In only four cases were additional bacteria grown: *Staphylococcus aureus* (*n* = 2), *Streptococcus pyogenes* (*n* = 1), *Streptococcus agalactiae* (*n* = 1). There was little difference in sputum culture results between admissions where picornaviruses were the sole finding and where another virus was detected.

Antibiotics were used in 94% of admissions when no viral infection was detected and in 82% to 84% of admissions when a virus was detected.

Testing for C‐reactive protein was performed in 63% of all admissions. One‐third of results were greater than or equal to 50mg/L, these higher levels being more likely for admissions with an identified viral infection.

## DISCUSSION

Our retrospective study of respiratory admissions for children with severe CP highlights an important burden of acute respiratory illness from community‐acquired viral infections. Reduced frequency of hospitalization for respiratory illness during periods of restricted social contact, implemented to reduce transmission of COVID‐19, mirrored trends in the wider population and underlines the importance of community viral transmission.^16^ The contribution of bacterial infection was less clear.

In children with severe CP, acute presentation with respiratory deterioration is multifactorial. Although aspiration is regarded as an important cause of acute respiratory illness, in this study it was difficult to quantify the relative contributions of aspiration and other components of respiratory pathology in individual respiratory presentations.^4^, ^17^ Aspiration can occur as a significant acute event, supported by radiological evidence of pneumonia. Alternatively, chronic primary and/or secondary aspiration can lead to exacerbations of chronic suppurative lung disease/bronchiectasis. However, investigations to diagnose bronchiectasis can be problematic in children with severe CP.^18^ Parenchymal abnormalities on chest radiographs can be difficult to assess where there is significant thoracic spine and rib cage asymmetry. High resolution CT to diagnose bronchiectasis typically require anaesthesia and were infrequently performed in our cohort. Only eight hospitalized children had chest CT, with three children confirmed to have bronchiectasis. Lower airway culture is recommended but was not always possible to obtain in our cohorts. Sputum collection is challenging in children who do not spontaneously expectorate and who may struggle to follow instructions, bronchoalveolar lavage requires general anaesthesia with associated risks and costs, while oropharyngeal swabs lack sensitivity for detection of lower airway pathogens,^19^ and detection of bacteria in the upper airway does not infer causation in lower airway pathology.^20^ Assessments for aspiration and gastro‐intestinal reflux disease are also difficult.^21^ Impedance probes provide evidence for gastro‐oesophageal reflux but not direct evidence of aspiration of the reflux. Isotope tests such as salivagram may document saliva aspirated into the lung, but the significance of this finding is uncertain, and milk scans are probably poorly sensitive outside the neonatal period. In essence, our study data provided little hard evidence for the presence or absence of chronic aspiration, suppurative lung disease, and bacterial infection in many individual children, although the presence of gram‐negative bacilli such as *Pseudomonas aeruginosa* may be indicative of underlying lung disease.^22^, ^23^ Their pathological role in acute presentations, however, remains to be clearly defined.

Restrictive lung disease, largely caused by thoracic kyphoscoliosis and muscle weakness, is another important component of respiratory pathology in children with severe CP. The mechanical disadvantage of the respiratory muscles posed by severe kyphoscoliosis and decreased chest wall compliance causes increased respiratory effort, decreased vital capacity, unequal lung ventilation, and increased risk for progressive respiratory failure. Chronic respiratory failure, manifested by hypoxia and/or hypoventilation, may be compounded by upper airway obstruction, particularly in the setting of low upper airway muscle tone.^24^ The mainstay of treatment is non‐invasive ventilation, initially during sleep, with or without the addition of a higher concentration of inspired oxygen.

Our study supports the important contribution of viral infection as a trigger for exacerbations in children who are at risk of having poor pulmonary reserve as a result of weakness or poorly co‐ordinated cough resulting in inefficient secretion clearance, restrictive lung disease, and/or chronic suppurative lung disease. In an earlier Australian study of children with non‐cystic fibrosis bronchiectasis, exacerbations were accompanied by positive viral status in 48%.^25^ Positive viral status was associated with increased odds of hospitalization, severe cough, hypoxia, fever, chest signs, and elevated C‐reactive protein levels.^25^ Similarly, positive viral status was reported in 40% of hospitalizations in our study, or in 48% of hospitalizations where respiratory viral screening was performed. There was no evidence to support a difference in severity of illness based on viral status, although high levels of inflammatory markers were more common in admitted children with evidence of viral infection. If viral infections are typically associated with increased airway secretions, the study raises questions around whether additional assistance with airway clearance and greater ventilatory support may be more important priorities than antibiotic treatment. Currently, prompt initiation of antibiotic therapy appears to be the commonly recommended and accepted approach.^26^

Although most viral infections were suppressed in hospitalized children during the lockdown period, there was increased detection of picornaviruses. This finding is consistent with literature reports of the persistence of rhinovirus infections.^27^ An intra‐household surveillance study showed that prolonged nasopharyngeal COVID‐19 ribonucleic acid persistence beyond the acute infection phase was frequent in adults quarantined at home during the first epidemic wave and that this was often associated with co‐infection with rhinovirus.^28^ The increased risk of transmission of rhinovirus to children during periods of social restriction, particularly when those children had higher care needs, provides a possible explanation for our findings.

There are some limitations in interpreting these study findings. The study only included children with CP functioning in GMFCS levels IV and V and hospitalizations at a single tertiary paediatric centre. The results may not be generalizable to other persons with severe neurological impairment or to other hospitals. It is also possible that presentations to the emergency department and hospital admission patterns may have been affected by additional factors during the COVID‐19 pandemic, although children's hospitals were not affected by the same extreme pressures experienced by adult services. The children who had sputum cultures were more frequently those with more severe illness requiring intensive care so may not be representative of the whole cohort.

This study suggests that more information is needed to better understand the contribution of multiple comorbidities to the respiratory presentation of individual children with severe CP. Given the lack of evidence to confirm lung pathology caused by aspiration, the high risk of restrictive lung disease and upper airway obstruction, and the frequency of viral pathology, are we making assumptions when we manage exacerbations based on non‐cystic fibrosis bronchiectasis guidelines? There is a need to better manage chronic respiratory disease in the outpatient setting and to treat exacerbations based on consideration of all respiratory comorbidities and family priorities. Many questions remain unanswered. What tools might help define specific pathophysiologies that will inform decisions around which children will derive most benefit from different interventions? How do we optimize use of chest CT clinically? Might low‐dose rapid CT protocols safely define structural lung disease? If bronchiectatic changes are detected, do current management guidelines apply in these children?^29^ What is the role for molecular viral studies and inflammatory markers and how might the results inform management, including use of antibiotics? Can we rely on sputum culture collected by ad hoc methods and are there practical ways to better sample for bacterial infection? Should antibiotics be delayed until there is further evidence to support the use of an appropriate microbial agent? Seeking answers to these questions will underpin our future research agenda.

In conclusion, this study highlights that viral infections are an important precipitant for acute respiratory illness and hospitalization in children with severe CP. It raises the question of whether better understanding of each child's respiratory disease and specific pathophysiology would provide opportunities for more personalized management.

## Supporting information


**Figure S1:** Flow diagram showing selection of study cohorts for the pre‐lockdown, lockdown, and post‐lockdown periods.

**Table S1:** Bacteria and viruses identified during respiratory admissions across periods.

## Data Availability

The data that support the findings of this study are available from the corresponding author upon reasonable request.
