# Supplementary figures and images for: Respiratory admissions and impact of COVID‐19 lockdowns for children with severe cerebral palsy
Source: Dev Med Child Neurol. 2025 May 8;67(12):1582–9. doi: 10.1111/dmcn.16346 (PMC12618963; doi:10.1111/dmcn.16346)

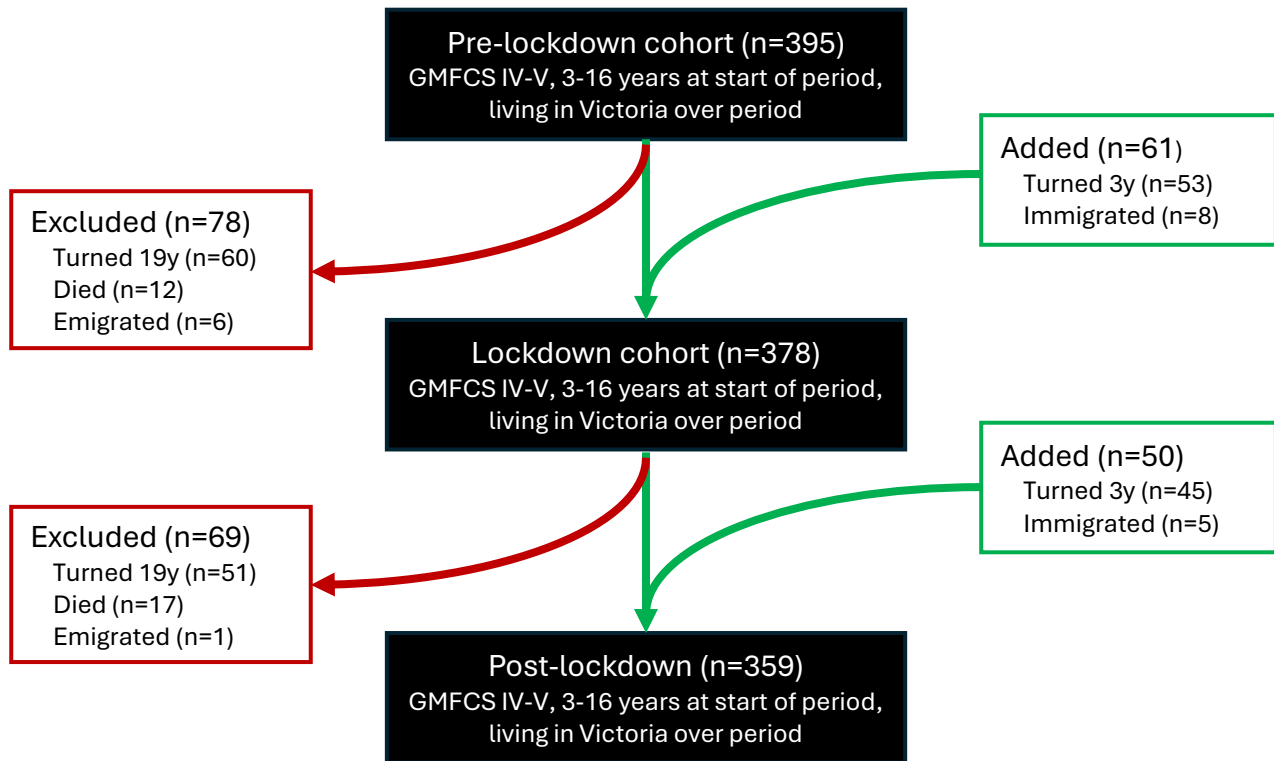

Supplement: Supplementary file 1 — Figure S1: Flow diagram showing selection of study cohorts for the pre‐lockdown, lockdown, and post‐lockdown periods. [file DMCN-67-1582-s002.pdf]
