# Supplementary material for: Respiratory admissions and impact of COVID‐19 lockdowns for children with severe cerebral palsy
Source: Dev Med Child Neurol. 2025 May 8;67(12):1582–9. doi: 10.1111/dmcn.16346 (PMC12618963; doi:10.1111/dmcn.16346)
Supplement: Supplementary file 2 — Table S1: Bacteria and viruses identified during respiratory admissions across periods. [file DMCN-67-1582-s001.docx]

Table S1. Bacteria and viruses identified during respiratory admissions across periods

|  | Pre-lockdown  n (n/146*100) | Lockdown  n (n/91*100) | Post-lockdown  n (n/170*100) | Total  n |
| --- | --- | --- | --- | --- |
| **VIRAL SCREEN** |  |  |  |  |
| Rhinovirus | 7 (4.8) | 13 (14.3) | 39 (22.9) | 59 |
| Human parainfluenza virus | 11 (7.5) | 3 (3.3) | 15 (8.8) | 29 |
| Enterovirus | 1 (0.7) | 10 (11.0) | 16 (9.4) | 27 |
| Respiratory syncytial virus | 7 (4.8) | 1 (1.1) | 13 (7.6) | 21 |
| Human metapneumovirus | 8 (5.5) | 1 (1.1) | 8 (4.7) | 17 |
| Influenza virus A or B | 7 (4.8) | 0 (0.0) | 10 (5.9) | 17 |
| Adenovirus | 2 (1.4) | 0 (0.0) | 8 (4.7) | 10 |
| Parechovirus | 2 (1.4) | 0 (0.0) | 0 (0.0) | 2 |
|  |  |  |  |  |
| **SPUTUM CULTURE** |  |  |  |  |
| *Pseudomonas aeruginosa* | 29 (19.9) | 15 (16.5) | 28 (16.5) | 72 |
| *Klebsiella pneumoniae* | 1 (0.7) | 6 (6.6) | 7 (4.1) | 14 |
| *Staphylococcus aureus* | 4 (2.7) | 3 (3.3) | 3 (1.8) | 10 |
| *Streptococcus pyogenes* | 3 (2.0) | 0 (0.0) | 3 (1.8) | 6 |
| *Escherichia coli* | 2 (1.4) | 0 (0.00 | 2 (1.2) | 4 |
| *Haemophilus influenzae* | 1 (0.7) | 2 (2.2) | 0 (0.0) | 3 |
| *Haemophilus haemolyticus* | 0 (0.0) | 2 (2.2) | 1 (0.6) | 3 |
| Mixed gram negative | 1 (0.7) | 0 (0.0) | 2 (1.2) | 3 |
| *Haemophilus parainfluenzae* | 0 (0.0) | 1 (1.1) | 1 (0.6) | 2 |
| *Moraxella catarrhalis* | 0 (0.0) | 1 (1.1) | 1 (0.6) | 2 |
| *Enterobacter cloacae* | 2 (1.4) | 0 (0.0) | 0 (0.0) | 2 |
| *Stenotrophomonas maltophilia* | 1 (0;7) | 1 (1.1) | 0 (0.0) | 2 |
| *Acinetobacter sp* | 1 (0.7) | 1 (1.1) | 0 (0.0) | 2 |
| *Streptococcus agalactiae* | 1 (0.7) | 0 (0.0) | 0 (0.0) | 1 |
| *Proteus miribalis* | 1 (0.7) | 0 (0.0) | 0 (0.0) | 1 |
| *Morganelli morganii* | 1 (0.7) | 0 (0.0) | 0 (0.0) | 1 |
| *Achromobacter xylosoxidans* | 0 (0.0) | 0 (0.0) | 1 (0.6) | 1 |
| *Serratia marcescens* | 0 (0.0) | 0 (0.0) | 1 (0.6) | 1 |
